# Enlarged Kidney

**Published:** 1884-09

**Authors:** William Pepper

**Affiliations:** Provost of and Professor of the Theory and Practice of Medicine in the University of Pennsylvania


### ENLARGED KIDNEY, PROBABLY SARCOMA.

### A CLINICAL LECTURE DELIVERED AT THE HOSPITAL OF THE UNIVERSITY OF PENNSYLVANIA.

BY WILLIAM PEPPER, M. D., LL. D.
Provost of and Professor of the Theory and Practice of Medicine in the University of Pennsylvania.

REPORTED BY WILLIAM H. MORRISON, M. D.

GENTLEMEN—I have not yet had an opportunity of obtaining this man's history, and shall ask your attention to his account of himself. His age is 47. Nine years ago he had a slight attack of hæmaturia, but after that had no trouble until three years ago, at which time, that is, in the early part of 1881, he had a stoppage of his water for four days. During this time, he says that he did not pass a drop of anything. He was not seen by a physician and no catheter was passed. The stoppage was accompanied with great pain. At the end of four days he discharged large amounts of clotted blood, and after this passed blood more or less for a year. The presence of blood in the urine was not constant, but spells of hæmaturia, lasting from ten days to two weeks, would occur at intervals of from two to four weeks. A month is, he thinks, the longest period that blood was absent from the urine during 1881. When these attacks occurred he had sharp pains running toward the groin, around the bladder and into the penis, and also had pain in the renal regions. Between the spells of hæmaturia he had no particular pain. He was at this time serving as night watchman of a cotton mill, and was not particularly exposed. He has never had chills and fever. This is an important point, for, as you are aware, the relations between malaria and hæmaturia are curious and interesting. This man has lived and worked in a central part of the city and has never suffered from ague. He is unable to trace the attacks of hæmaturia to anything that he did. They came at all seasons of the year.

In February, 1882, one year ago, he applied to the dispensary, and since then there has been no blood in the urine. He seemed to be relieved by the use of large doses of the astringent salts of iron. Two years ago he weighed 140 pounds; he now does not weigh more than 110 pounds. No blood has been passed for a year. A recent examination of the urine by Dr. William E. Hughes shows no tube-casts, but a small amount of albumen, and in the sediment a small amount of pus cells, the amount of pus being scarcely sufficient to account for the amount of albumen in the supernatant liquid. The specific gravity is normal and the reaction acid. Examination of the blood reveals nothing but slight anæmia.

In addition to this, we learn that in 1875, while on the police force, he had an attack of hæmatemesis. This came on after no unusual exposure, and while he was apparently in perfect health. He had lain down one afternoon, and, after sleeping for about ten minutes, was awakened with his mouth full of blood. He then began to vomit large quantities of blood. This vomiting continued for four days, and he says that he lost at least a "bucketful" of blood. He has never been a drinking man and never met with any injury.

Turning to the examination of the belly, we find it prominent in the umbilical region. This is more distinctly observed to the right of the median line. On palpation, a mass is immediately recognized below the margin of the ribs on the right side. It extends from the ribs down to the anterior-superior spinous process of the ilium, where its lower margin is felt. This body extends outwards to the median line, but it does not seem to be a continuous body, for there is a vertical groove into which I can introduce the tips of my fingers. This mass might readily be taken for a tumor growing from the liver, or for a portion of the right lobe of the liver, if it were not for this deep groove to which I have just referred. Is it possible that this is the transverse fissure of the liver, and this mass on the right an enlarged right lobe, while that to the left is a greatly hypertrophied left lobe? Or is this a mass loosely connected with the liver? Or is it distinct from the liver, pushing the enlarged organ to the left.

Let us first consider what the conditions are that could have caused the vomiting of blood which he tells us took place eight years ago. As you know, hæmatemesis is common in ulcer of the stomach, in cirrhosis of the liver and in malignant disease of the stomach; but in the latter affection, the blood is usually vomited in small quantities (the matters ejected being simply tinged with blood) and in the form of coffee-ground material. Copious hæmatemesis is rarely due to malignant disease. There are no symptoms justifying the diagnosis of ulcer of the stomach. It would, therefore, be altogether plausible to assume that he had cirrhosis of the liver and that the engorged gastric veins suddenly relieved themselves by the hæmatemesis. This is a symptom which not rarely occurs in the course of cirrhosis and may be one of the first to attract the attention of the patient. We might say that this man had an enlarged cirrhotic liver, but this would not account for the attacks of hæmaturia which recurred for twelve months, closing with February, 1882. These would point to some serious disease of the kidneys themselves. This is borne out by the pain in the renal regions, radiating along the course of the ureters and into the penis, which recurred with the attacks of hæmaturia, so that when we, to-day, find in the urine the evidences of pyelitis (pus and a small amount of albumen without tube-casts), it leads us to suspect that we have to deal with some serious affection of the kidneys, perhaps an impacted calculus, and we are forced to consider whether or not this mass on the right side may not be the right kidney greatly enlarged and pushing the liver to the left and downward. The supposition that this is the right kidney is strengthened by the history of the case, by the general feel and character of this mass, and by the fact that I can succeed in forcing my finger to a considerable depth between it and what seems to be the enlarged right lobe of the liver. When I examine for a similar enlargement on the left side, I fail to find it. I find the left lobe of the liver projecting downwards. The spleen does not appear to be enlarged.

From this examination I am compelled to suspect that we have here a great cystic kidney or a sarcoma of the right kidney. This mass is easily grasped by the fingers. It extends into the lumbar region and is slightly movable. I can force it backwards until it bulges out posteriorly. It seems to be more movable than a tumor of the kidney should be, but in all other respects it corresponds with that supposition.

It is of the greatest importance that we should know the exact nature of this mass, for if it is an enlarged kidney, the question of extirpating it must be considered. I propose, therefore, to introduce an exploring needle into the centre of this body to see if it contains liquid. That failing, I shall, by means of a delicate instrument, remove a shred of tissue for microscopical examination. If it proves to be an enlarged kidney, I shall call a consultation to consider the advisability of removing it. Taking advantage of the relaxation of the abdominal walls, I have succeeded in getting my finger between this mass and the liver above. There is a groove continuous with the one I

before pointed out. The right lobe lies above the margin of the ribs. This still further confirms the supposition that this mass is connected with the right kidney, and it justifies the exploratory puncture which I propose to make in order to determine more positively its exact character. If this be a cyst of the kidney, with, perhaps, a calculus obstructing the ureter, the organ should be removed. Even if it be a sarcoma, I should be in favor of the removal of the kidney.

The man's general condition seems to be pretty fair. The only complication would be the existence of secondary disease of the liver. Although we may presume that this mass has exerted a displacing effect upon the liver, yet we find that that organ extends so far above the margin of the ribs and so far below that we must conclude that the liver is enlarged. If we were sure that this was not merely a congestive enlargement, we should not think of any interference with this mass.

I shall try to persuade him to stay in the hospital for a few days, in order that we may make the exploratory puncture.

